# Assessing the performance of public–private partnerships in non-communicable disease management with a mixed-methods approach

**DOI:** 10.1038/s41598-025-23266-7

**Published:** 2025-11-11

**Authors:** Mehrdad Salehi, Amir Ghasemian, Aboalfazl Marvi, Ehsan Mousa Farkhani, Sara Karimi, Leili Alizamani, Javad Moghri, Seyed Saeed Tabatabaee

**Affiliations:** 1 Student Research Committee, Mashhad University of Medical Sciences, Mashhad, Iran; 2 Department of Epidemiology, School of Health, Mashhad University of Medical Sciences, Mashhad, Iran; 3 Student Research Committee, School of Public Health, Hormozgan University of Medical Sciences, Bandar Abbas, Iran; 4 Social Determinants of Health Research Center, Mashhad University of Medical Sciences, Mashhad, Iran; 5 Department of Management Sciences and Health Economics, School of Health, Mashhad University of Medical Sciences, Mashhad, Iran

**Keywords:** Non-communicable diseases, Public-Private partnerships, Health system performance, Primary healthcare, Mixed-methods, Iran, Diseases, Health care, Health occupations

## Abstract

Non-communicable diseases (NCDs) are a major global health challenge, particularly in low- and middle-income countries (LMICs) with constrained health systems. Public-Private Partnerships (PPPs) are increasingly utilized to improve access and efficiency in primary healthcare. This study evaluates the performance of public and outsourced healthcare centers in managing NCDs and identifies systemic and operational barriers to effective PPP implementation. A sequential mixed-methods design was employed, combining quantitative analysis of NCD-related key performance indicators (KPIs) from the Integrated Health System (2018–2022) using SPSS v26 with qualitative insights from semi-structured interviews with 16 healthcare providers, analyzed via conventional content analysis in MAXQDA 2020. Public centers consistently outperformed outsourced centers in screening, diagnosis, and disease management KPIs. Two primary barrier categories were identified (1): managerial challenges, including ineffective outsourcing, poor coordination, inadequate incentive structures, and weak monitoring; and (2) operational challenges, such as staff turnover, suboptimal health information systems, fragmented service delivery, and inadequate infrastructure. While PPPs can enhance service delivery in resource-constrained settings, their effectiveness in NCD management is limited by structural and governance issues. Policy reforms focusing on robust oversight, intersectoral collaboration, and health system investments are critical. These findings provide valuable insights for optimizing PPPs in similar LMIC contexts.

**Supplementary Information:**

The online version contains supplementary material available at 10.1038/s41598-025-23266-7.

## Introduction

Non-communicable diseases (NCDs) are the leading cause of global morbidity and mortality, accounting for nearly 75% of all deaths and over 60% of the global disease burden^1^. The World Health Organization (WHO) reports that over 80% of premature NCD deaths—those occurring before age 70—occur in low- and middle-income countries (LMICs), highlighting their disproportionate burden in these regions^2^. In Iran, NCDs account for 83.5% of deaths and 78.1% of the national disease burden, significantly impacting population health^1,3,4^. This burden is exacerbated by an epidemiological transition driven by aging populations, lifestyle changes, and the rising prevalence of chronic conditions such as diabetes and cardiovascular diseases^5^. These diseases place considerable financial strain on health systems in LMICs, where limited resources often restrict access to prevention and treatment, leading to broader socioeconomic consequences^6,7^.

Managing NCDs is a critical challenge for health systems in many LMICs. Public-Private Partnerships (PPPs) have emerged as innovative strategies to expand primary healthcare (PHC) coverage and enhance service delivery. Evidence from South Asia suggests that PPPs can improve access to care, though concerns about equity and sustainability persist^8^. Similarly, Brazil’s Previne Brasil program demonstrates how financing mechanisms can strengthen PHC, yet gaps in NCD management remain^9^. A recent systematic review underscores that successful PPPs in LMICs rely on robust governance, effective quality assurance, and sustainable financing^10^. These global insights provide a valuable framework for assessing Iran’s experience with PPPs.

Previous research on PPPs in Iran has largely focused on financing, infrastructure, or broader PHC reforms (e.g., Marvi et al., 2024; Doshmangir et al., 2019)^11,12^. However, there is limited empirical evidence on their role in NCD management. This study addresses this gap by evaluating PPP models for NCD care in Bandar Abbas, a city with a high burden of diabetes and cardiovascular disease. By examining this context, the study contributes novel insights to the Iranian literature and informs global discussions on PPPs.

PHC is widely recognized as a cost-effective and equitable approach to tackling NCDs. In line with WHO global action plans, Iran has implemented an “essential package” for NCD prevention and control, targeting cardiovascular diseases, diabetes, chronic respiratory conditions, and preventable cancers. This package was integrated into PHC through the Health Transformation Plan (HTP), a major health reform initiative^13^. Despite these efforts, structural barriers—such as fragmented service delivery, inadequate infrastructure, and weak health information systems—continue to impede effective PHC implementation^14^.

Introduced in 2016, the HTP aimed to expand human resources, improve accessibility, and strengthen referral systems. Key initiatives included extending first-level services to urban, rural, and nomadic populations, enhancing the family physician program, promoting self-care and oral health, and implementing national programs for both communicable and non-communicable diseases^12^. Outsourcing through PPPs was a key strategy to address public sector capacity constraints^15,16^.

Global evidence suggests that outsourcing can enhance accessibility, quality, and efficiency, but its effectiveness varies^17^. While PPPs have succeeded in high-income countries like the United Kingdom and the United States^18,19^, outcomes in middle-income countries such as Jordan, Tunisia, and Lebanon are mixed. In countries like Egypt, Iran, and Afghanistan, PPPs are relatively new, and evidence on their impact is still emerging^20,21^.

In Iran, the private sector has increasingly contributed to PHC services, particularly in urban and peri-urban areas, focusing on first-level NCD care. This study evaluates the performance of PPP-based NCD programs in southern Iran, specifically in Bandar Abbas. By providing context-specific evidence, it aims to inform national health policy and contribute to the global discourse on PPPs in healthcare.

## Methods

### Study design

This study employed a sequential mixed-methods approach comprising two phases.

#### Quantitative

The quantitative phase involved a descriptive cross-sectional analysis of non-communicable disease (NCD) program performance indicators extracted from the Integrated Health System (IHS) database, the primary health services registry at Hormozgan University of Medical Sciences, for public and outsourced healthcare centers in Bandar Abbas. The qualitative phase used content analysis to explore healthcare providers’ perspectives and identify barriers to implementing NCD programs in these centers.

#### Qualitative

Quantitative data were extracted from the IHS in late 2023, while qualitative interviews were conducted in early 2024. This sequential design provided a comprehensive overview of program implementation from 2018 to 2022, capturing disruptions caused by the COVID-19 pandemic (2020–2021) and the subsequent recovery phase. To mitigate potential recall bias in retrospective reporting, we triangulated IHS data and recruited providers with continuous employment at the centers throughout the study period.

## Study setting and participants

**Fig. 1:**
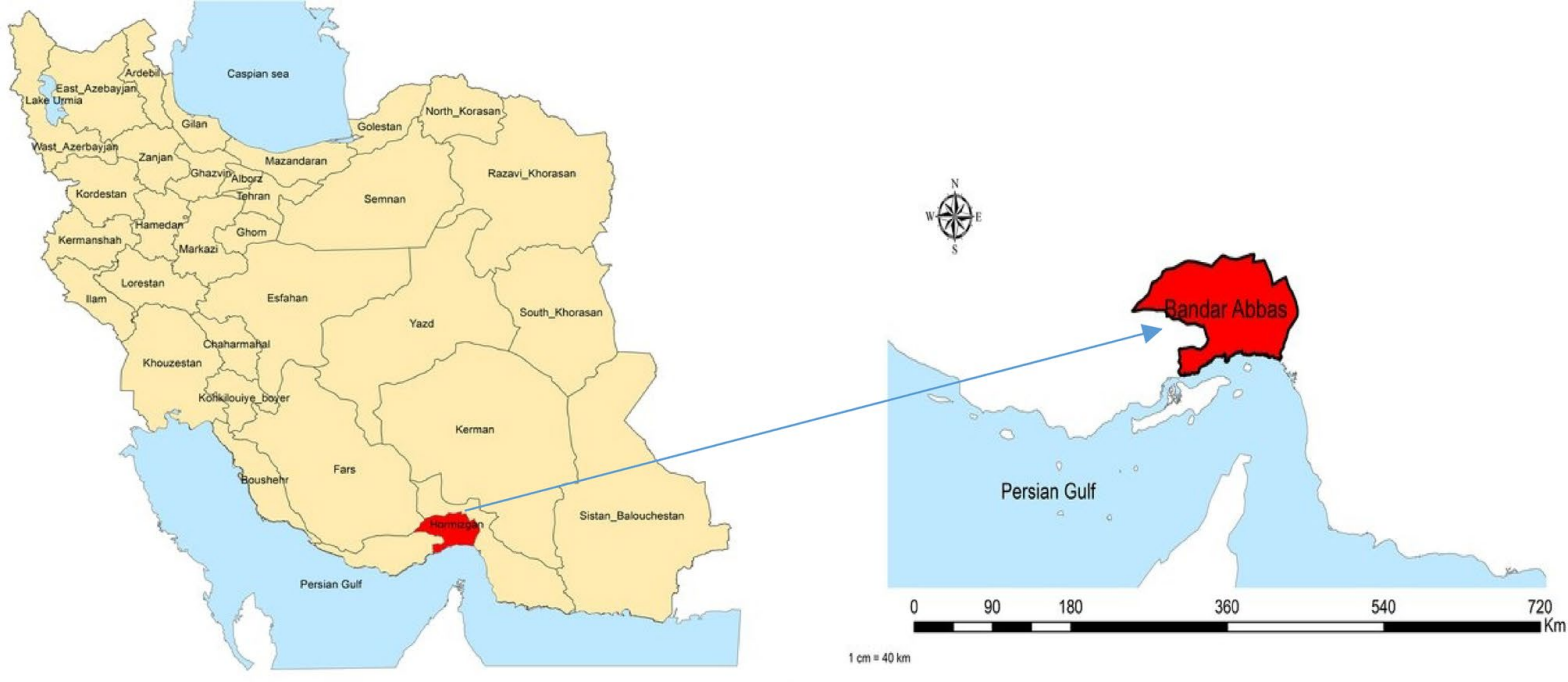
Location of Bandar Abbas, the capital of Hormozgan Province, southern Iran. Adapted from Nikpour et al.^22^, reproduced with permission from Wiley (License No. 6120650090321).

The study was conducted in Bandar Abbas, Hormozgan Province, southern Iran, across four public and four outsourced comprehensive health centers (CHCs) located in adjacent areas for comparative analysis. Participants included 16 family physicians and community health workers responsible for delivering NCD programs. We used purposive sampling with a maximum variation strategy to ensure diverse perspectives, selecting participants based on gender, academic background, professional role, and center type (public or outsourced). Participant demographics are reported in Table 6. To address recall bias for services delivered between 2018 and 2022, participants were encouraged to reference documented experiences, supported by IHS records where possible, during interviews conducted in 2024. A study area map illustrating the geographical location of Bandar Abbas, the capital city of Hormozgan Province, is presented in Fig. 1.

## Eligibility criteria

For the quantitative phase, all four outsourced CHCs in Bandar Abbas were included after coordination with the Vice Chancellor for Health at Hormozgan University of Medical Sciences. Public CHCs were selected based on similarities in geographic location, organizational characteristics (e.g., service delivery, human resources, infrastructure, and equipment), population coverage, and contextual factors (e.g., economic, social, and cultural). Centers not meeting these criteria were excluded. Each CHC served a comparable population, ranging from 13,000 to 37,000 individuals, with public centers covering approximately 91,000–106,000 residents and outsourced centers covering 79,000–92,000 residents during the study period (2017–2021 IHS records). Adults aged ≥ 30 years, the primary target for NCD programs, comprised approximately 48–50% of the population in both center types (~ 52,000 in public and ~ 45,000 in outsourced centers in 2017), ensuring valid performance comparisons.

For the qualitative phase, participants required at least two years of experience in NCD program implementation at the selected centers and willingness to participate. Those unwilling to join or continue interviews were excluded.

## Data collection

Data were collected using a mixed-methods approach.

### Quantitative data collection

For the quantitative phase, key performance indicators (KPIs) for NCD programs (diabetes, hypertension, dyslipidemia, and stroke risk assessments) were extracted from CHCs using a checklist (Appendix 1) and the IHS database over a five-year period (2018–2022). Data quality was verified by flagging and removing implausible values (e.g., duplicates or outliers) and cross-checking missing entries against national KPI dashboards. A sample data extraction form is provided in Appendix 1 for transparency. Data were analyzed using SPSS version 26.

### Qualitative data collection

For the qualitative phase, semi-structured in-depth interviews were conducted in Persian from May to June 2024, each lasting 30–60 min. The interview guide was developed based on a literature review and research team discussions, refined after three pilot interviews (Appendix 2). Interviews were conducted by the first author (MS), a doctoral student with primary healthcare experience, and a health expert (SK). Participants received consent forms and the interview guide in advance, and interviews were scheduled at their convenience. Study objectives were explained at the start of each session, with probing questions (e.g., “Could you elaborate?” or “Can you provide an example?”) used for clarity. Interviews were recorded with consent, and non-verbal cues were noted by an assistant. Data collection ceased after Interview 13 when data saturation was reached, with three additional interviews conducted for confirmation. The SQUIRE checklist was used to guide reporting^23^.

### Data analysis

#### Quantitative data analysis

Quantitative data were analyzed using descriptive statistics (mean and standard deviation) to compare performance indicators between public and outsourced CHCs. As the study included all four outsourced and four matched public CHCs, it represented a census, making inferential analysis secondary. However, supplementary independent-samples t-tests were conducted for selected KPIs (2018–2022), reporting p-values and 95% confidence intervals (Supplementary Tables S1–S4). Standardized mean differences (SMDs) were calculated to assess effect sizes. Indicators were standardized per 1,000 adults aged ≥ 30 years to account for population size differences, with implausible values excluded and missing data validated against national KPI dashboards.

#### Qualitative data analysis

Qualitative data were analyzed concurrently with collection. Interviews were transcribed verbatim in Word 2019 by MS and an assistant, then coded inductively in MAXQDA 2020 by MS and AM. Transcriptions were read word-by-word to extract meaning units, which were condensed, coded, and grouped into subcategories based on similarities and differences. Subcategories were then organized into main themes. The research team reviewed and refined the findings collaboratively.

### Study trustworthiness

The trustworthiness of findings was assessed using Lincoln and Guba’s criteria^24^. Credibility was ensured through maximum variation sampling and active team engagement throughout the study. Transferability was supported by a detailed description of the research process and unbiased reporting of participant quotes. Coherence was achieved by sharing transcribed interviews with participants for verification and having two researchers dedicate sufficient time to coding. Confirmability was maintained through team reviews of the findings.

#### Ethical approval and consent **for participation**

The study was approved by the Medical Ethics Committee of Mashhad University of Medical Sciences (IR.MUMS.FHMPM.REC.1402.204). Participants received oral and written information about the study and provided written informed consent. Ethical principles of autonomy, non-maleficence, privacy, and data protection were upheld, in accordance with the Helsinki Declaration^25^.

## Results

### Quantitative

We compared 15 performance indicators related to diabetes, hypertension, dyslipidemia, and cardiovascular risk assessment between governmental and outsourced Community Health Centers (CHCs). We present descriptive statistics (mean ± SD and 95% CI), standardized per 1,000 adults aged ≥ 30 years, in Tables 1, 2, 3 and 4. Overall, governmental CHCs exhibited greater continuity and control in non-communicable disease (NCD) care, particularly for diabetes and cardiovascular risk assessment. In contrast, outsourced CHCs showed stronger performance in earlier years but experienced a declining trend thereafter.

To complement descriptive comparisons, we conducted independent-samples t-tests for selected key performance indicators (KPIs) between governmental and outsourced CHCs from 2018 to 2022 (Supplementary Tables S1–S4). Although most differences were not statistically significant (*p* > 0.05), standardized mean differences (SMDs) consistently favored governmental CHCs in diabetes control and dyslipidemia detection. These findings suggest that governmental CHCs maintained more stable performance, while outsourced CHCs faced sustainability challenges.

The catchment populations of governmental and outsourced CHCs were demographically similar. Governmental CHCs served 91,000–106,000 residents, while outsourced CHCs served 79,000–92,000. Adults aged ≥ 30 years comprised approximately 48–50% of the population in both models, ensuring a fair comparison of performance indicators. We provide detailed year-by-year values for all 15 indicators in Table 5.

**Table 1:** Comparison of diabetes detection rates between governmental and outsourced CHCs (mean ± SD, 95% CI, SMD), 2018–2022.

Year	Governmental (mean ± SD, 95% CI)	Outsourced (mean ± SD, 95% CI)	SMD (Gov − Out)
2018	0.5 ± 0.3% (0.1–0.8)	0.4 ± 0.3% (0.1–0.6)	0.23
2019	0.2 ± 0.1% (0.1–0.2)	0.3 ± 0.2% (0.1–0.4)	−0.59
2020	0.2 ± 0.1% (0.1–0.3)	0.2 ± 0.2% (0.1–0.4)	−0.59
2021	0.1 ± 0.0% (0.0–0.1)	0.1 ± 0.1% (−0.0–0.2)	−0.43
2022	0.1 ± 0.1% (0.0–0.2)	0.1 ± 0.1% (−0.0–0.2)	0.45

Footnote for Tables 1, 2, 3 and 4: SMD = Standardized Mean Difference; CI = Confidence Interval. Values standardized per 1,000 adults aged ≥ 30 years.

**Table 2:** Comparison of diabetes control rates between governmental and outsourced CHCs (mean ± SD, 95% CI, SMD), 2018–2022.

Year	Governmental (mean ± SD, 95% CI)	Outsourced (mean ± SD, 95% CI)	SMD (Gov − Out)
2018	51.7 ± 16.3% (33.2–70.2)	45.1 ± 48.1% (−2.1–92.3)	0.17
2019	57.3 ± 16.6% (41.0–73.5)	44.8 ± 12.7% (32.4–57.2)	0.84
2020	62.6 ± 20.6% (42.4–82.8)	87.2 ± 22.2% (62.1–112.3)	−1.16
2021	47.3 ± 22.0% (25.7–68.9)	75.0 ± 35.4% (26.0–124.0)	−1.07
2022	55.4 ± 14.6% (41.0–69.7)	40.3 ± 46.1% (−4.8–85.5)	0.44

**Table 3:** Comparison of hypertension detection rates between governmental and outsourced CHCs (mean ± SD, 95% CI, SMD), 2018–2022.

Year	Governmental (mean ± SD, 95% CI)	Outsourced (mean ± SD, 95% CI)	SMD (Gov − Out)
2018	0.7 ± 0.5% (0.2–1.2)	0.6 ± 0.4% (0.2–1.0)	0.17
2019	0.5 ± 0.2% (0.3–0.6)	0.6 ± 0.3% (0.3–0.8)	−0.51
2020	0.2 ± 0.2% (0.1–0.4)	0.3 ± 0.2% (0.1–0.5)	−0.19
2021	0.1 ± 0.1% (0.0–0.2)	0.1 ± 0.2% (−0.0–0.3)	−0.18
2022	0.1 ± 0.2% (−0.0–0.3)	0.1 ± 0.1% (−0.0–0.3)	0.14

**Table 4:** Comparison of dyslipidemia detection rates between governmental and outsourced CHCs (mean ± SD, 95% CI, SMD), 2018–2022.

Year	Governmental (mean ± SD, 95% CI)	Outsourced (mean ± SD, 95% CI)	SMD (Gov − Out)
2018	8.1 ± 5.3% (2.9–13.3)	7.6 ± 2.9% (4.7–10.4)	0.13
2019	8.1 ± 4.2% (4.0–12.2)	6.9 ± 4.2% (2.8–11.0)	0.29
2020	8.2 ± 3.1% (5.1–11.2)	4.9 ± 3.5% (1.5–8.3)	0.98
2021	16.7 ± 14.4% (2.6–30.8)	4.4 ± 3.5% (1.0–7.8)	1.17
2022	15.0 ± 9.0% (6.2–23.9)	4.5 ± 1.7% (2.8–6.2)	1.62

**Table 5:** Comparison of quality of care and outcome measures between private and governmental comprehensive health Centers.

Health Indicator	Center Type	2018	2019	2020	2021	2022
Screening for diabetes and high blood pressure through risk assessment care for heart and strokes (%)	Governmental	9.62	8.50	4.87	4.31	10.40
	Outsourced	11.29	9.66	5.06	4.05	9.55
	*Difference	−1.67	−1.16	−0.20	0.26	0.85
Diabetic Patient Detection (%)	Governmental	0.44	0.17	0.14	0.06	0.11
	Outsourced	0.34	0.22	0.24	0.09	0.05
	Difference	0.10	−0.05	−0.10	−0.03	0.06
Diabetes Prevalence Rate (%)	Governmental	3.93	3.99	4.04	3.89	3.99
	Outsourced	3.89	4.02	4.18	4.06	4.10
	Difference	0.05	−0.03	−0.14	−0.17	−0.11
Diabetic patients cared for by physician (%)	Governmental	3.57	10.79	7.74	3.33	6.26
	Outsourced	3.50	9.80	3.47	2.50	6.16
	Difference	0.07	0.99	4.27	0.83	0.10
Diabetes Control (%)	Governmental	58.11	65.81	59.20	51.32	60.54
	Outsourced	29.51	43.65	63.24	52.00	27.20
	Difference	28.60	22.17	−4.04	−0.68	33.34
Diabetic patients have tested HbA1c (%)	Governmental	3.18	5.49	1.38	1.05	1.06
	Outsourced	1.15	2.28	0.31	0.00	0.64
	Difference	2.03	3.21	1.07	1.05	0.42
Diabetic patients with normal HbA1c (%)	Governmental	21.21	41.18	51.61	41.67	52.00
	Outsourced	30.00	38.10	33.33	0.00	23.08
	Difference	−8.79	3.08	18.28	41.67	28.92
Pre diabetic Patient Detection (%)	Governmental	7.59	7.80	4.80	3.91	8.39
	Outsourced	5.16	4.40	5.78	6.56	4.66
	Difference	2.43	3.40	−0.98	−2.65	3.73
Pre diabetic Patient Care (%)	Governmental	0.00	0.00	87.69	91.92	91.00
	Outsourced	0.00	0.00	100.00	93.13	85.91
	Difference	0.00	0.00	−12.31	−1.21	14.00
Blood Pressure Detection (%)	Governmental	0.69	0.41	0.21	0.10	0.14
	Outsourced	0.58	0.51	0.27	0.11	0.10
	Difference	0.11	−0.10	−0.05	−0.01	0.04
Blood Pressure Prevalence Rate (%)	Governmental	5.78	6.02	6.09	5.86	5.99
	Outsourced	5.99	6.37	6.50	6.28	6.36
	Difference	−0.22	−0.34	−0.41	−0.42	−0.37
Blood Pressure Control (%)	Governmental	73.88	74.00	78.92	79.90	82.47
	Outsourced	77.36	82.33	88.89	93.30	85.42
	Difference	−3.48	−8.34	−9.97	−13.39	−2.95
Hyperlipidemia patients Detection Rate (%)	Governmental	8.39	8.29	8.49	18.60	12.60
	Outsourced	6.58	5.98	3.12	3.40	4.89
	Difference	1.82	2.32	5.37	15.20	7.71
People who have done risk assessment and are referred immediately (%)	Governmental	0.26	0.43	0.52	0.28	0.51
	Outsourced	0.14	0.09	0.08	0.15	0.25
	Difference	0.12	0.34	0.43	0.13	0.25
People who have done a risk assessment and have been referred immediately and have been under care (%)	Governmental	92.31	90.00	100.00	85.71	93.55
	Outsourced	100.00	100.00	100.00	100.00	91.67
	Difference	−7.69	−10.00	0.00	−14.29	1.88

*Difference = Governmental - Private(outsourcing).

**Fig. 2:**
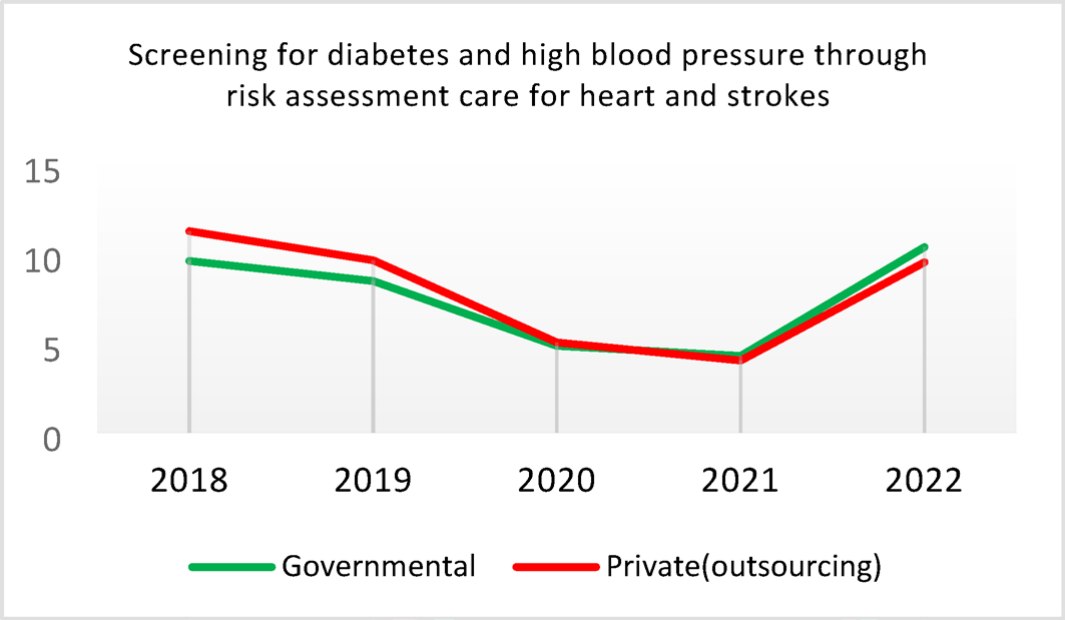
Screening for diabetes and high blood pressure through risk assessment care for heart and strokes (%).

**Fig. 3:**
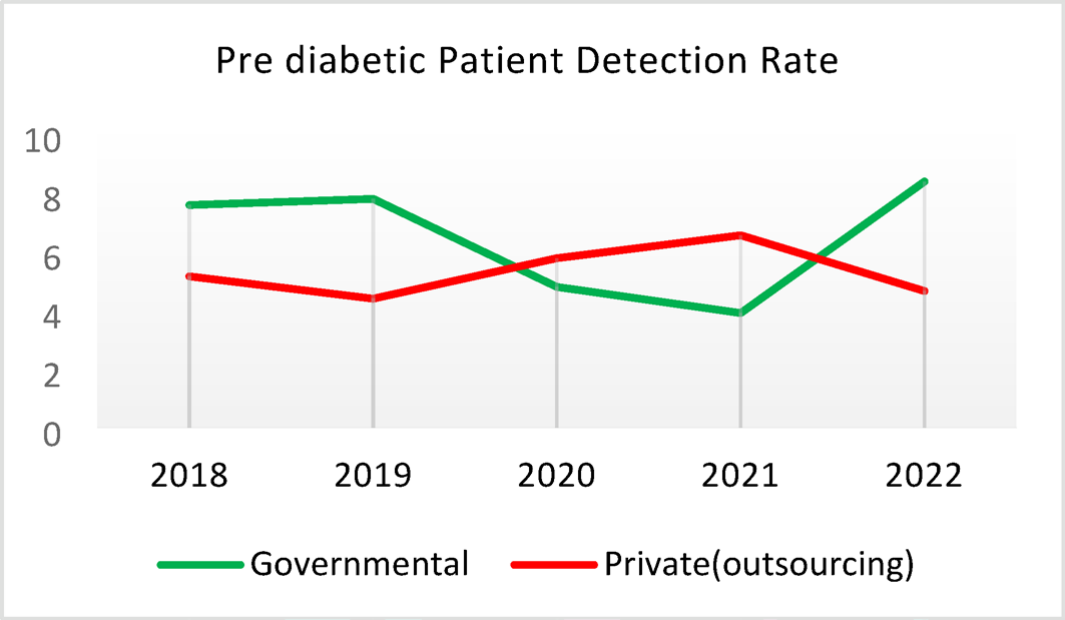
Diabetic Patient Detection (%).

**Fig. 4:**
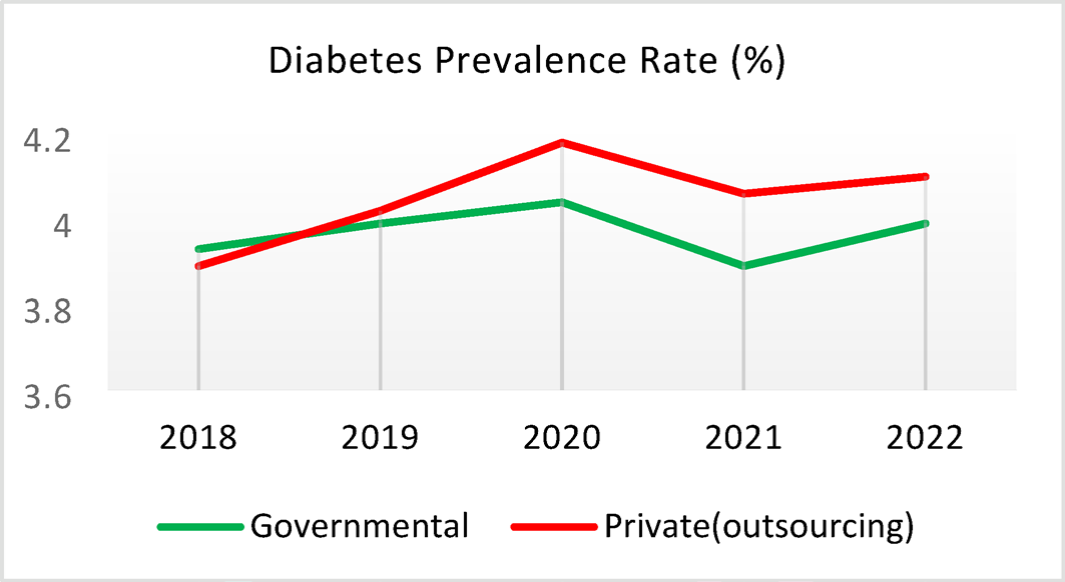
Diabetes Prevalence Rate (%).

**Fig. 5:**
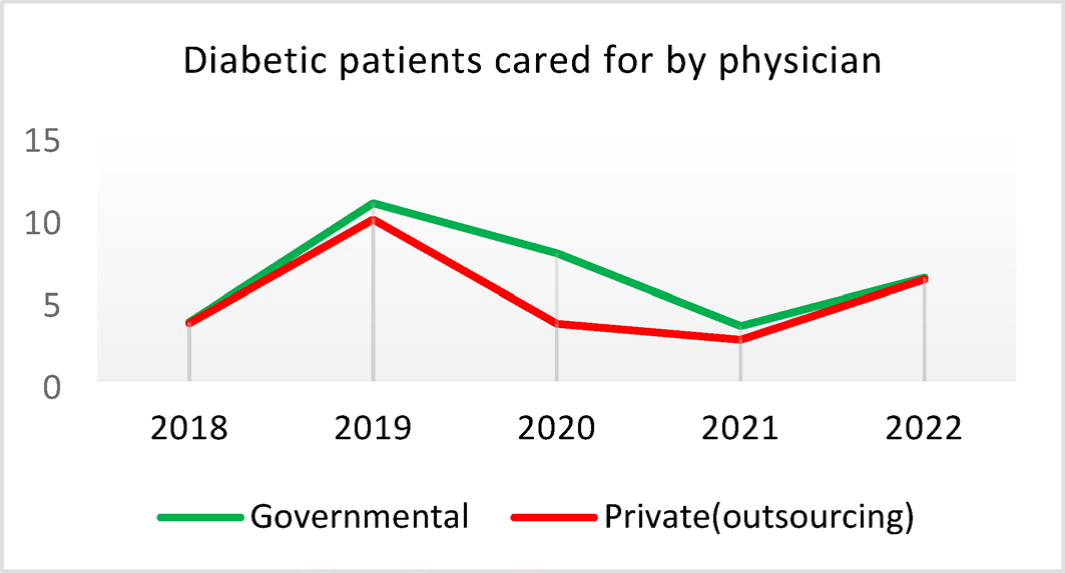
Diabetic patients cared for by physician (%).

**Fig. 6:**
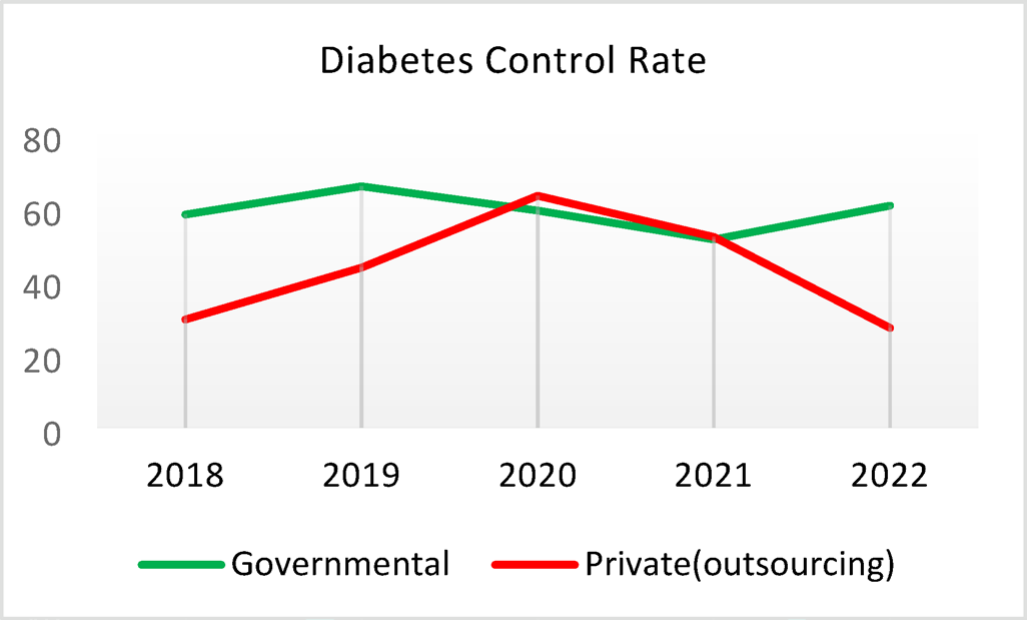
Diabetes Control (%).

**Fig. 7:**
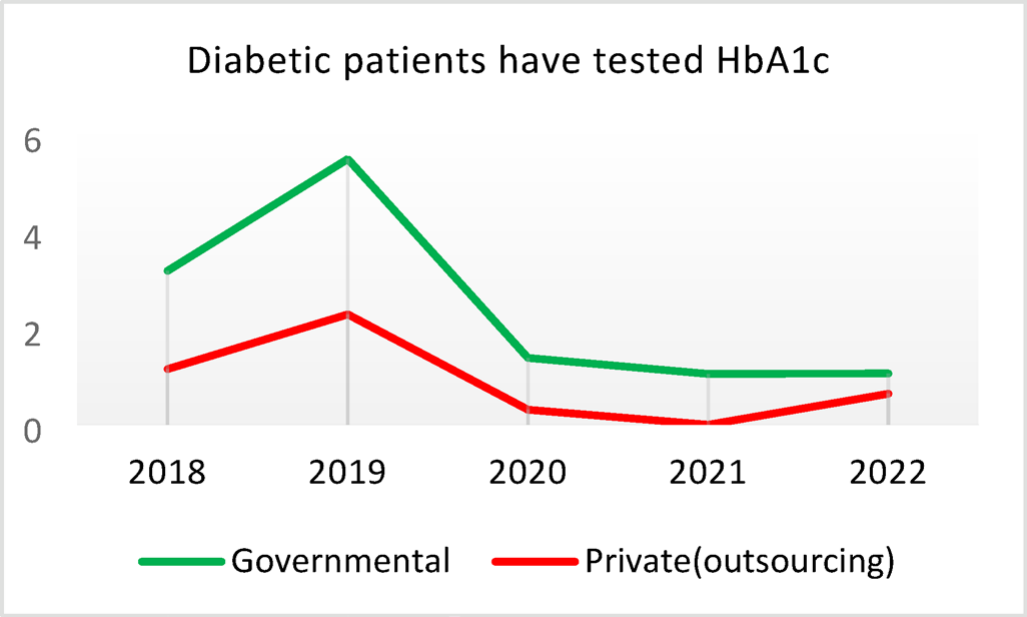
Diabetic patients have tested HbA1c (%).

**Fig. 8:**
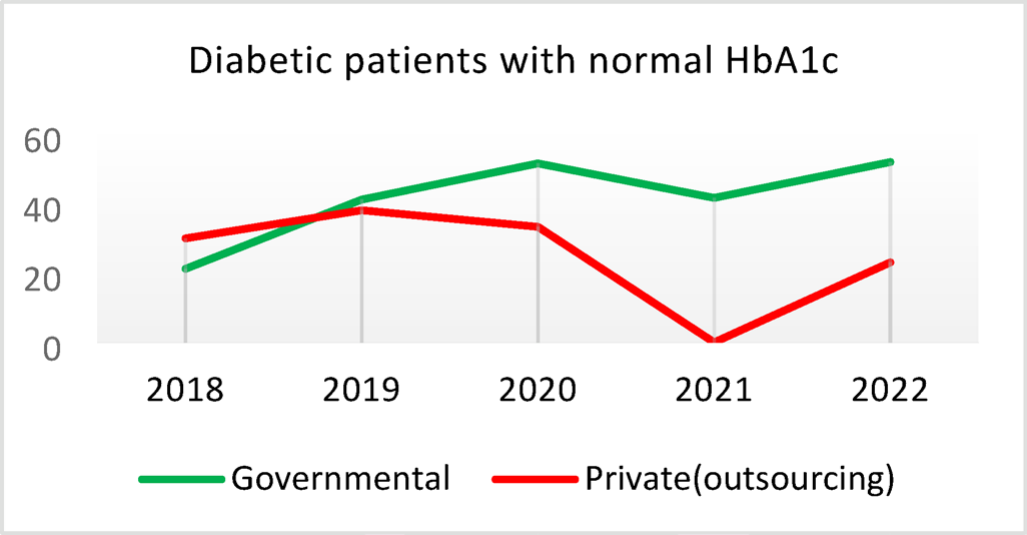
Diabetic patients with normal HbA1c (%).

**Fig. 9:**
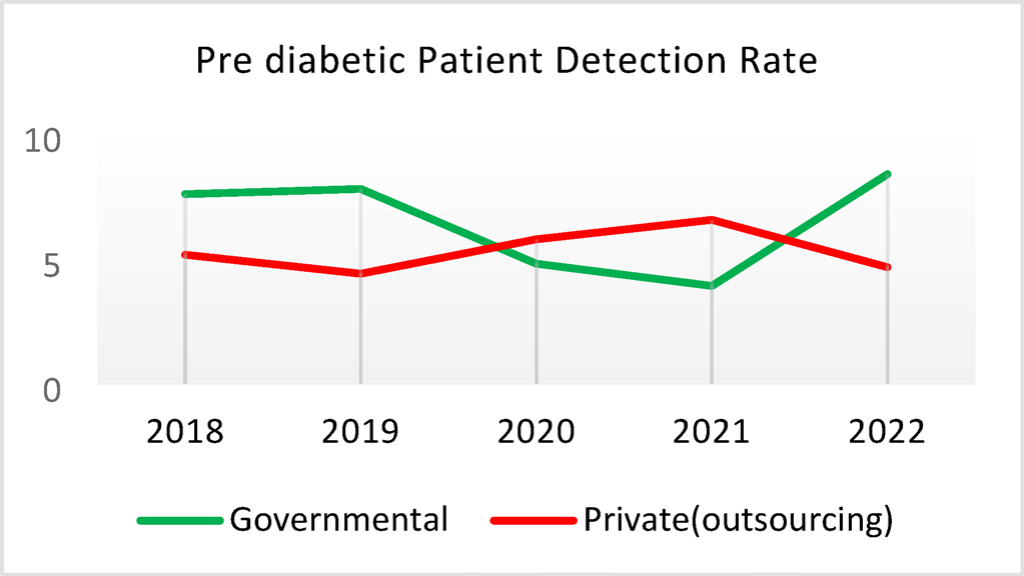
Pre diabetic Patient Detection (%).

**Fig. 10:**
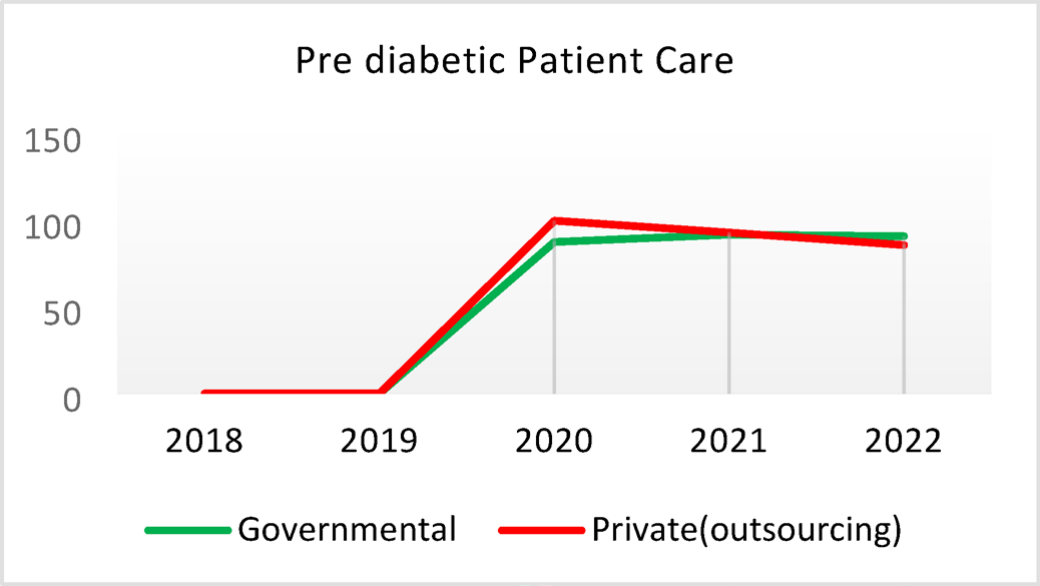
Pre diabetic Patient Care (%).

**Fig. 11:**
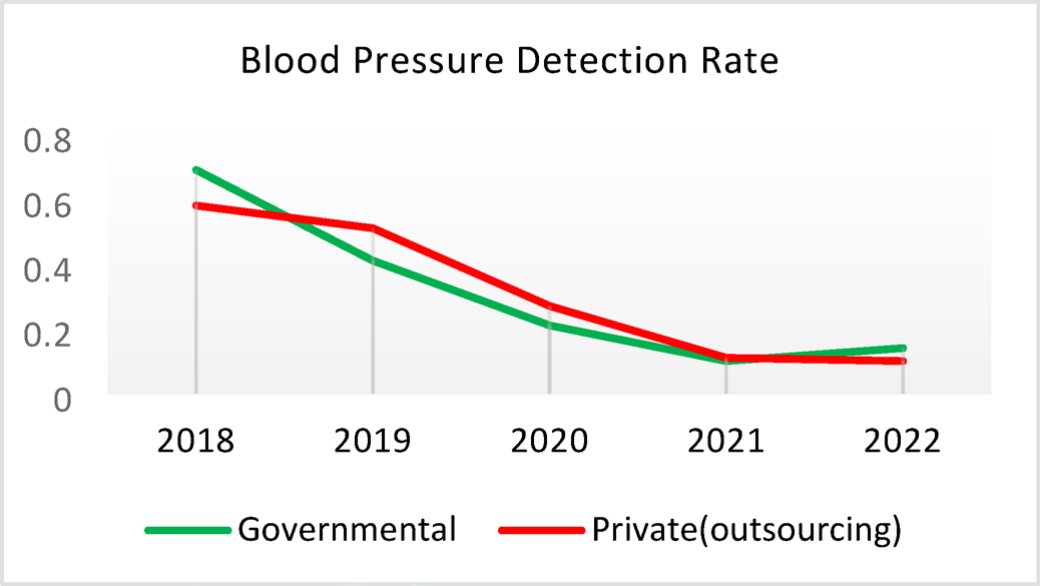
Blood Pressure Detection (%).

**Fig. 12:**
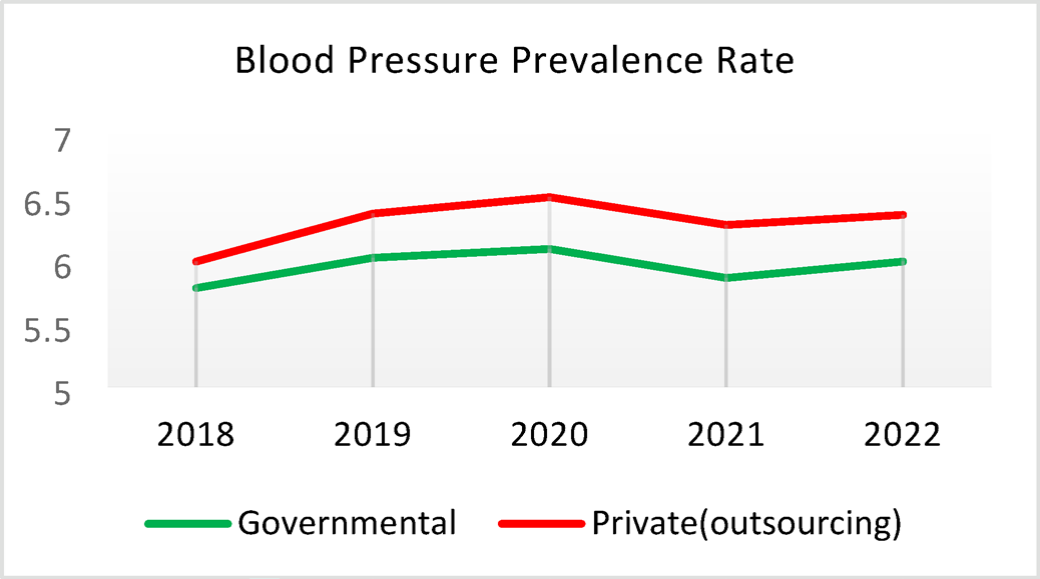
Blood Pressure Prevalence Rate (%).

**Fig. 13:**
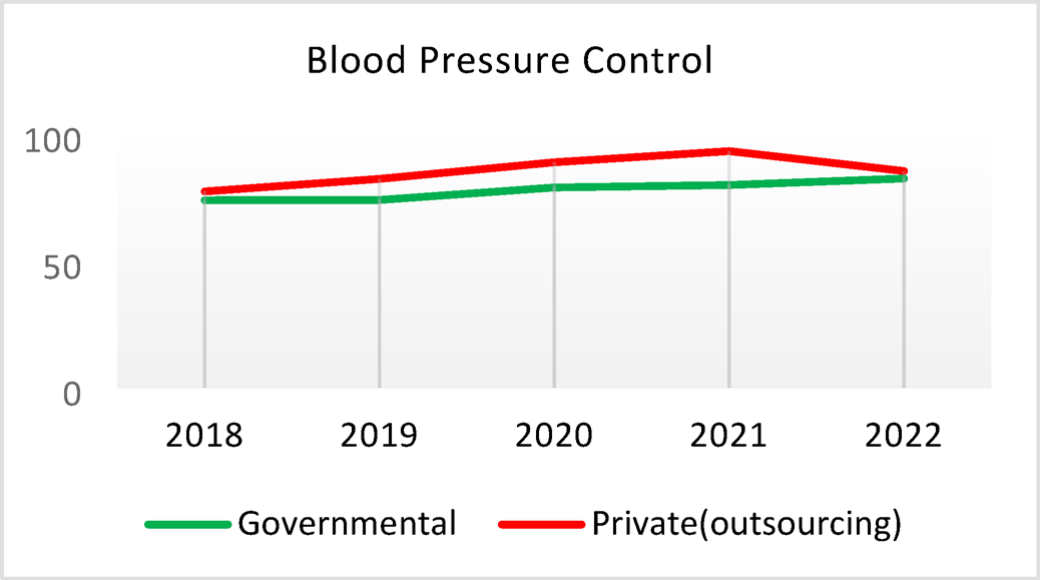
Blood Pressure Control (%).

**Fig. 14:**
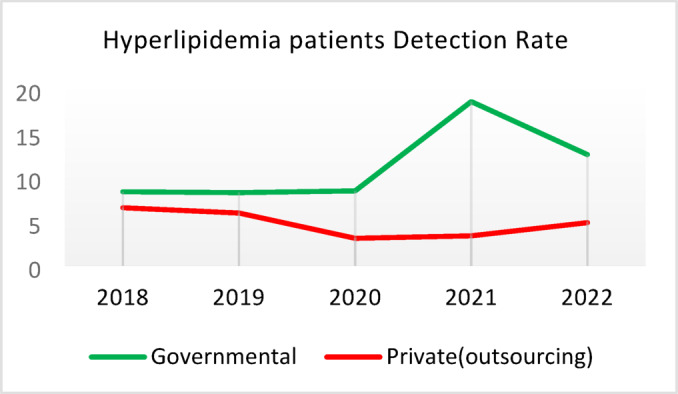
Hyperlipidemia patients Detection Rate (%).

**Fig. 15:**
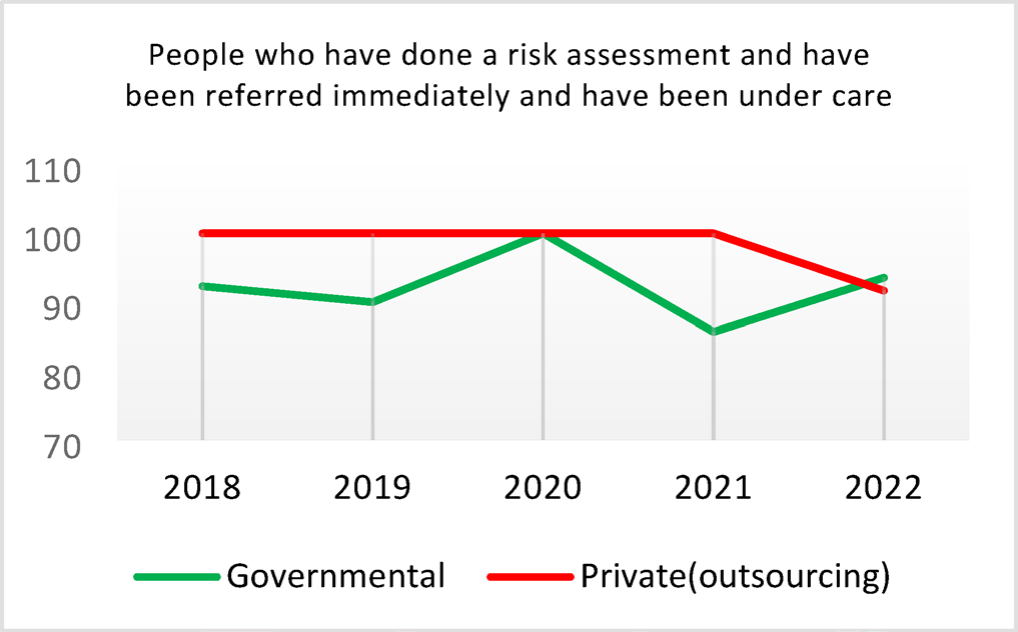
People who have done risk assessment and are referred immediately (%).

**Fig. 16:**
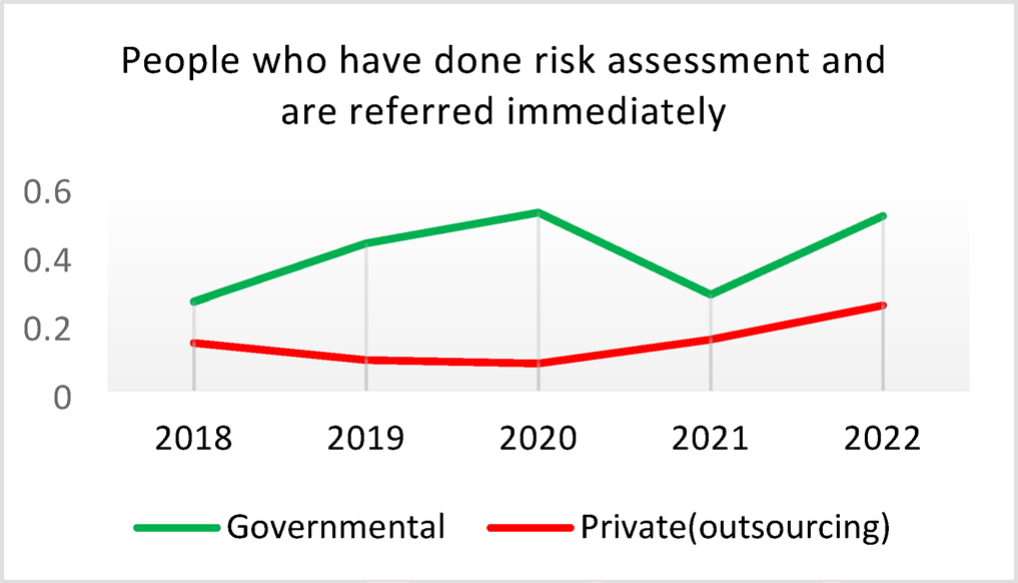
People who have done a risk assessment and have been referred immediately and have been under care (%).

1. **Screening for Diabetes and Hypertension**: Both types of CHCs showed similar trends. Outsourced CHCs performed slightly better at the start of the review period, but by the end, governmental CHCs surpassed them (Fig. 2).
2. **Diagnosis of Diabetic Patients**: Both CHC types exhibited significant fluctuations in this indicator. Governmental CHCs performed slightly better at the beginning and end of the period, while outsourced CHCs outperformed them in the middle (Fig. 3).
3. **Prevalence of Diabetes**: Both CHC types maintained relatively stable diabetes prevalence rates over the 5-year period, with annual fluctuations of less than 0.2%. Outsourced CHCs reported slightly higher prevalence, likely due to increased screening and diagnosis rates in 2019 and 2020 (Fig. 4).
4. **Diabetic Patients Under Physician Care**: Both CHC types saw a decline in physician care for diabetic patients from 2019 to 2021. However, this rate slightly increased in both models in 2022 (Fig. 5).
5. **Diabetes Control**: Governmental CHCs outperformed outsourced CHCs in this indicator. Although both types experienced fluctuations, those in outsourced CHCs were more pronounced (Fig. 6).
6. **Diabetic Patients Tested for HbA1c**: Governmental CHCs achieved higher HbA1c testing rates than outsourced CHCs. Both types saw a decline in testing from 2018 to 2022, with a more significant decrease in outsourced CHCs (Fig. 7).
7. **Diabetic Patients with Controlled HbA1c (Normal)**: Patients under governmental CHC care had higher rates of controlled HbA1c compared to those in outsourced CHCs. Outsourced CHCs showed a decreasing trend until 2021, while governmental CHCs maintained more stable performance (Fig. 8).
8. **Diagnosis of Prediabetes**: Both CHC types showed significant fluctuations in this indicator. Governmental CHCs performed slightly better at the beginning and end of the period, while outsourced CHCs excelled in the middle (Fig. 9).
9. **Care for Prediabetes**: Neither CHC type recorded care for prediabetic individuals in the integrated health information system (*IHS*) in 2018 and 2019. In 2020, outsourced CHCs outperformed governmental CHCs, but governmental CHCs demonstrated more consistent and less variable performance overall (Fig. 10).
10. **Diagnosis of Hypertension**: Governmental CHCs performed better at the start of the review period. Both CHC types experienced a significant decline in hypertension diagnosis from 2019 to 2021. In 2022, governmental CHCs improved their diagnosis rate by 0.04%, while outsourced CHCs saw a 0.01% decline (Fig. 11).
11. **Prevalence of Hypertension**: Both CHC types maintained relatively stable hypertension prevalence rates over the 5-year period, with annual fluctuations of less than 0.3%. Outsourced CHCs reported slightly higher prevalence due to increased screening and diagnosis in 2019 and 2020 (Fig. 12).
12. **Control of Hypertension**: Both CHC types showed an upward trend in hypertension control, with outsourced CHCs performing slightly better until 2021. In 2022, outsourced CHCs exhibited a downward trend, while governmental CHCs continued to improve (Fig. 13).
13. **Identification of Hyperlipidemia Patients**: Governmental CHCs consistently outperformed outsourced CHCs in this indicator, peaking in 2021. In 2022, governmental CHCs experienced a slight decline, while outsourced CHCs showed a gradual increase in identification rates in 2021 and 2022 (Fig. 14).
14. **Risk Assessment and Immediate Referral**: Governmental CHCs showed an upward trend in this indicator from 2018 to 2020, while outsourced CHCs exhibited a declining trend. In 2021, outsourced CHCs reversed this trend and showed improvement into 2022. Governmental CHCs, however, experienced a decline in 2021 but improved in 2022 (Fig. 15).
15. **Risk Assessment**,** Immediate Referral**,** and Follow-Up Care**: From 2018 to 2021, outsourced CHCs maintained a consistent 100% rate, while governmental CHCs showed fluctuations. In 2020, both CHC types achieved 100% care for referred patients. In 2022, governmental CHCs improved their rate by 1.88% compared to outsourced CHCs (Fig. 16).

### Qualitative

We engaged 16 service providers, including physicians, health care experts, and midwives, from both governmental and outsourced Comprehensive Health Centers (CHCs). Table 6 presents the participants’ demographic characteristics.

**Table 6:** Demographic information of the study participants.

Variable		*N*	%
Gender	Male	4	25
	Female	12	75
Academic degree	MD	12	75
	Bachelor	4	25
Positions	Family physicians	4	25
	Community health workers	12	75
Place of service	Government Centers	8	50
	Outsourced Centers	8	50
Average age(years)	33.1		
Job experiences (years)	8.8		

After analyzing the qualitative data, we categorized the challenges faced by service providers in non-communicable disease (NCD) programs into two main themes and eight sub-themes: managerial challenges (outsourcing, collaboration, payment mechanisms, and monitoring and evaluation) and operational challenges (human resources, health information, service delivery, and infrastructure). Table 7 summarizes these findings.

**Table 7:** Challenges facing NCDs programs in comprehensive health centers.

Category	Subcategory	Items
**Managerial Challenges**	Inefficient outsourcing of health services	• Weak communication between private companies and the public sector • Poor accountability of public companies regarding their commitments • Lack of familiarity of private companies with the functions of the health sector • Inadequate and weak supervision of private companies’ performance
	Weak intersectoral collaboration and coordination	• Weak community engagement and participation • Insufficient use of health volunteers’ capacities • Limited utilization of health philanthropists’ resources • Poor intersectoral collaboration and partnerships • Weak utilization of private sector capabilities • Ineffective intrasectoral collaboration within the health and medical domains
	Inadequate and non-incentivizing payment mechanisms	• Discrepancies in payment levels between public and private sector employees • Delays and irregularities in payments by outsourcing companies • Lack of proper implementation of performance-based payment systems • Non-payment of motivational and incentive items • Workload misalignment with payment levels in Comprehensive Health Centers • Absence of standardized criteria for payment benchmarks
	Ineffective monitoring and evaluation systems	• Unreasonable and unrealistic goal-setting • Weak execution of supportive monitoring and fostering communication between supervisors and supervisees • Focus on service quantity over quality • Inadequate consideration of human resources, equipment, and facilities during performance assessments • Insufficient attention to cultural and social factors in the community being evaluated • Neglect of service recipients’ feedback during assessments
**Operational Challenges**	Shortage of qualified and stable human resources	• Disproportion of human resources with the designated population in centers • Shortage of staff and high workload for human resources • Staff turnover and low retention in centers • Low job security among private sector employees • Low motivation among both public and private sector employees • Weakness in implementing training and retraining programs for employees • Lack of appropriate welfare facilities for employees at the workplace • Difference between private and public sector employees in using the welfare facilities and amenities of the university
	Weaknesses in health information systems	• Inefficiency of the ‘IHS’ system in service delivery • Limited access of health caregivers to the ‘IHS’ system for patient registration and follow-up • Lack of integration between public and private doctors for shared patient information in defined populations • Reliance on paper-based, Excel, and web-based data collection methods • Failure to update and complete system information regularly by staff
	Inefficiencies in service delivery processes	• Limited scope of services provided in Comprehensive Health Centers, especially laboratory and pharmacy services • Insufficient attention to the socio-economic and cultural aspects of communities in service delivery • Weak implementation of educational programs, campaigns, and promotion of self-care and health literacy • Public preference for treatment-focused approaches and weak collaboration in receiving preventive and care services • Lack of proper incentives to increase public participation, especially in screening programs • Low public awareness of non-communicable disease risk factors • Incomplete implementation of the patient referral system
	Aging infrastructure and insufficient medical equipment	• Shortage of adequate physical spaces for service delivery in Comprehensive Health Centers • Lack of basic facilities for patient use • Insufficient equipment for service provision, such as glucose test strips and blood pressure monitors • Poor quality of existing equipment in centers • Shortage of computers for service registration and follow-up • Internet and telephone issues affecting patient follow-up and consultation efforts

### Managerial challenges

Participants identified poor management in areas such as outsourcing, inter-sectoral and intra-sectoral collaboration, community participation, payment mechanisms, and monitoring and evaluation as significant barriers to providing NCD services. They noted that these managerial weaknesses created operational obstacles in primary health care centers, particularly affecting private-sector providers.

### Outsourcing

Participants highlighted that private companies often lack familiarity with the health sector’s technical functions, exhibit weak accountability due to poor communication with the health department, and face inadequate supervision of their performance. One interviewee stated: *“In my opinion*,* private companies do not have good communication with the health department*,* and coordination between these companies is very weak. This has led to a decline in the accountability of private companies towards their commitments to staff and the performance of centers. These companies are not very familiar with the operations of the health sector. In my view*,* they are just manpower providers and are not well-managed or supervised in terms of paying salaries. Moreover*,* supervision over them is also weak.”* (P3, Family Physician).

### Collaboration

Effective primary health care, especially for NCDs, relies on inter-sectoral coordination and community participation. Participants identified barriers including weak inter-sectoral coordination to leverage other organizations’ capacities, poor intra-sectoral coordination within the health department (particularly in communication and patient referral systems), and limited community involvement. One interviewee remarked: *“As a service provider*,* one of our problems is the weak cooperation with other organizations under our coverage. In our opinion*,* the management and executive offices should create this coordination. If this coordination is managed properly*,* services will improve*,* and there will be better opportunities to compensate for resource shortages in centers.”* (P8, Community Health Visitor (CHV)). Another added: *“I think one of the things we should focus on is community participation. We are not making good use of this capacity. Voluntary health organizations in local areas or even health philanthropists*,* who may even be patients under our care*,* could facilitate the care process for non-communicable disease patients.”* (P4, CHV).

### Payment mechanism

Participants emphasized that payment mechanisms significantly influence program execution and service delivery. They identified issues such as the lack of an effective performance-based payment system, delays in payments (especially by outsourced companies due to inadequate supervision), misalignment between workload and compensation (particularly for private-sector staff), and the absence of standardized payment criteria or adequate financial and non-financial incentives. One interviewee stated: *“We were hired as private staff. In my opinion*,* performance-based payment is not implemented. The difference between my earnings and a public sector worker’s is significant*,* and it has reduced my motivation. Why should the payment method differ so much?”* (P9, CHV). Another noted: *“Companies do not cooperate well. Resources are provided to them*,* but they do not pay our salaries on time. On the other hand*,* they do not offer any incentives or reward schemes for program implementation. I wish this could happen*,* and that payments could improve so we can work with more motivation.”* (P6, CHV).

### Monitoring and evaluation

Participants underscored the critical role of monitoring and evaluation in aligning activities with program goals but identified several weaknesses, including unrealistic goal setting, an overemphasis on service quantity over quality, insufficient consideration of centers’ capacities and contextual factors during performance assessments, and inadequate supportive monitoring. They also stressed the need for better communication between monitors and staff. One interviewee explained: *“In my opinion*,* the goals and indicators are idealistic*,* and it is impossible to achieve them with the current workload*,* personnel*,* and resources. Goals should be set logically and in consideration of the center’s conditions; otherwise*,* quality will be affected.”* (P5, Family Physician). Another added: *“One of the problems with monitoring and evaluation is the lack of proper communication between the monitor and the monitored. Monitoring should be supportive and have a corrective approach. Monitors only point out our weaknesses and rarely guide us. This perspective needs to be corrected.” “In my opinion*,* when evaluating center performance*,* the capacities of the centers*,* the type of population*,* contextual factors*,* and human resources should be considered. Simply ranking centers based on indicators is not enough and takes us away from focusing on quality.”* (P11, CHV).

### Operational challenges

Participants identified significant operational challenges in delivering NCD services, including issues related to human resources, health information management, service delivery, and infrastructure and equipment. They noted that these challenges are closely tied to managerial weaknesses.

### Human resource

Participants highlighted a shortage of staff, high turnover, low motivation, and job insecurity, particularly among private-sector employees. They also pointed to poor-quality training programs and inadequate welfare facilities, especially in outsourced CHCs. One interviewee stated: *“A doctor at a Comprehensive Health Center has to see children*,* pregnant women*,* the elderly*,* teenagers*,* and non-communicable disease patients. The volume of services provided makes it difficult to give proper care to non-communicable disease patients and achieve their satisfaction.”* (P12, Family Physician). Another noted: *“We are always faced with a shortage of staff at the centers*,* and the workload is too heavy for us to dedicate sufficient time to the care of non-communicable disease patients.”* (P1, CHV). A third added: *“Health staff lack sufficient motivation. We don’t have good welfare facilities at the centers*,* and we cannot access university welfare services. Overall*,* health staff are less prioritized compared to employees in other health sectors. This has led to a decrease in our motivation.”* (P7, CHV). Finally, one interviewee remarked: *“I am a private sector employee; I have been working for several years but have not been hired by the public sector. They tell us to go through the labor department. But we work under the Ministry of Health. Why don’t they take action? We want to be hired and have job security.”* (P2, CHV).

### Health information

Participants identified inefficiencies in the Integrated Health System (*IHS*), restricted access to recording certain services, poor communication between family physicians and specialists, reliance on paper-based data collection and Excel files instead of the *IHS*, and incomplete or outdated patient records due to workload and staff shortages. One interviewee explained: *“One of our problems is the inefficiency of the IHS system. Many of the indicators and statistics we record cannot be extracted correctly*,* which is also a major challenge for management levels. They ask us for information using paper methods and Excel software. We have to enter information in multiple places. When do we have time to care for our patients?”* (P10, CHV). Another noted: *“As a health care provider*,* I am unable to register some of the services that are part of my duties*,* for example*,* for follow-ups of diabetic and hypertensive patients*,* it must have been at least a month since their last visit. If less than a month*,* the system won’t allow us to register. We should have access because we also have two-week follow-ups.”* (P16, CHV).

### Service delivery

Participants identified challenges in service delivery, including the lack of comprehensive services, failure to tailor services to the community’s economic, cultural, and social needs, rising laboratory and medication costs, weak promotion of self-care, low public awareness of NCD risk factors, a preference for specialist care, and incomplete implementation of the referral system. One interviewee stated: *“We don’t provide the required services for patients effectively at the centers. The services are not comprehensive. Our centers are supposed to be comprehensive health service centers*,* but we don’t have a laboratory or pharmacy*,* and sometimes we don’t even have blood glucose test strips. Patients come and ask why they should visit a Comprehensive Health Center. For non-communicable disease patients*,* they say*,* ‘You say my blood pressure is high*,* but what are you going to do for me? You refer me to a specialist*,* but we can go to the specialist ourselves. What do you do besides measure?’”* (P13, Family Physician). Another added: *“Many of the patients under our care have economic problems. They can’t even afford their medication or tests*,* and they don’t come for follow-up care. Services should be tailored to the economic and social conditions of the community.”* (P5, Family Physician). A third noted: *“One of our major problems in identifying and caring for non-communicable disease patients is the low public awareness about the health services provided at centers and about the risk factors for non-communicable diseases and self-care. We need to intervene in this area.”* Finally, one interviewee emphasized: *“We have a fundamental issue with the referral system. Referral for non-communicable disease patients is very important. We need information and actions taken by specialists*,* but the referral system is not fully implemented*,* and we cannot stay informed about the care of our patients. This is really important.”* (P3, Family Physician).

### Infrastructure and equipment

Participants highlighted inadequate physical space, insufficient facilities for patient comfort, and a lack of sufficient or high-quality equipment as barriers to delivering NCD services. One interviewee stated: *“Comprehensive Health Centers*,* in my opinion*,* need renovation and transformation. Many of them are not in good condition in terms of buildings and facilities. As a health care provider*,* I don’t have enough space to serve non-communicable disease patients. Patients need counseling and education*,* and this care requires appropriate physical space that ensures privacy and respects the dignity of patients. On top of that*,* some of these centers are rented*,* and we have to move*,* which makes it difficult for patients to visit regularly.”* (P11, CHV). Another noted: *“One of our problems is the lack of appropriate equipment to care for non-communicable disease patients. My colleagues and I at the Comprehensive Health Centers don’t have enough equipment like blood glucose test strips or blood pressure monitors for the number of patients we need to care for.”* (P12, Family Physician). A third added: *“As a health care provider*,* I have a specific population that I must invite for follow-ups and reminders to visit the center for care. I need a phone*,* but there is no phone in my room. In fact*,* our center has only one phone*,* which is in the reception area. I have to use my personal phone.”* (P14, CHV).

## Discussion

This mixed-methods study comprehensively evaluates the performance of governmental and outsourced primary health care (PHC) centers in managing non-communicable diseases (NCDs) in southern Iran, focusing on diabetes, hypertension, dyslipidemia, and cardiovascular risk assessment. By integrating quantitative performance indicators with qualitative insights, we document comparative performance and explore the systemic, managerial, and operational factors driving these outcomes. Framing our findings within the WHO health system building blocks framework provides a robust conceptual lens and highlights policy implications relevant to Iran and other low- and middle-income countries (LMICs) facing similar challenges.

### Quantitative findings

Our quantitative analysis reveals a nuanced pattern: outsourced PHC centers outperformed governmental centers in early screening and diagnosis during 2018–2020, but their performance declined thereafter, while governmental centers showed steady improvement and eventually surpassed outsourced centers. This trend reflects a common challenge in LMICs, where public-private partnerships (PPPs) deliver short-term gains but struggle to sustain them without robust oversight and reinvestment^26–28^. Similar patterns appear in India, Pakistan, and Bangladesh, where initial PPP-driven improvements waned due to inadequate monitoring and financing mechanisms^29–32^. The sharp decline in screening across both sectors during 2020–2021 aligns with global disruptions caused by the COVID-19 pandemic, which diverted resources from routine NCD management and restricted care access^33–36^. However, governmental centers demonstrated a stronger post-pandemic recovery, highlighting their integration with national programs and greater resource reallocation capacity^37^. Brazil’s public PHC systems, embedded within the Family Health Strategy, similarly exhibited resilience during COVID-19 compared to PPP arrangements vulnerable to supply chain and staffing disruptions^38^.

While diabetes and hypertension prevalence rates remained stable across both center types, outsourced centers reported slightly higher rates in some years, likely due to variations in catchment populations and reporting practices rather than actual disease burden differences^39^. More critically, governmental centers consistently achieved better disease control, particularly for diabetes, while outsourced centers showed a declining trend. This mirrors findings from Bangladesh and Egypt, where PPP programs struggled to maintain chronic care continuity due to weak oversight and financing irregularities^40–42^. In contrast, Brazil’s structured chronic disease models with multidisciplinary teams improved outcomes through enhanced continuity of care^28,42^.

Diagnostic performance also varied. HbA1c testing declined in both sectors but remained stronger in governmental centers, reflecting their more reliable laboratory networks and procurement systems^43–46^. Outsourced centers faced fragile procurement chains, a common issue in LMIC private-sector supply continuity. Similarly, governmental centers performed more consistently in prediabetes follow-up and risk identification, underscoring the value of institutional stability and trained staff^47^. Evidence from Sub-Saharan Africa and South Asia confirms that weak procurement and laboratory infrastructure undermine PPP performance.

Cardiovascular risk assessment further highlights these differences. Governmental centers aligned more closely with national guidelines and integrated NCD protocols, while outsourced centers excelled in immediate referrals and flexible follow-up, likely due to stronger community ties. However, both sectors struggled with long-term referral and counter-referral systems, a systemic issue observed in Iran, South Asia, and other LMICs^48,49^. These gaps emphasize the need for stronger referral pathways, shared health records, and multidisciplinary collaboration.

In summary, our quantitative findings suggest that while PPPs can expand access and drive initial improvements, governmental centers offer greater sustainability and continuity. International evidence reinforces that long-term PPP success requires integration into robust governance and national systems rather than reliance on short-term contracts.

### Qualitative findings

The qualitative findings complement and help explain these quantitative patterns by identifying governance, workforce, financing, service delivery, health information systems (HIS), and infrastructure as critical determinants of PPP performance. Outsourced centers were particularly challenged by unclear contracts, limited technical capacity of private providers, and insufficient public oversight. Comparable governance gaps have been observed in Pakistan and Bangladesh, where PPPs often prioritized service volume over quality because of weak contractual clarity and inadequate supervision^11,50,51^. Governmental centers, by contrast, benefited from stronger stewardship and closer integration into national programs, emphasizing the central role of state oversight in shaping PPP outcomes. Workforce instability was another critical issue, as outsourced centers reported staff shortages, high turnover, job insecurity, and low motivation, exacerbated by salary delays and the absence of performance-based incentives. These conditions undermined staff morale and continuity of care. Similar problems have been reported in India, where PPP staff employed on short-term contracts experienced higher attrition and lower job satisfaction, reinforcing the importance of job security, timely payments, and professional development opportunities^7,49,52,53^. In contrast, governmental centers offered more stable employment conditions that contributed to stronger long-term outcomes, highlighting the need for comprehensive incentive packages that include both financial and non-financial benefits.

Health information systems were also identified as a major weakness. Outsourced centers struggled with duplicate data entry, restricted access to integrated platforms, and limited interoperability, all of which increased workloads and reduced efficiency. These challenges resemble those reported in Kenya and Nigeria, where weak HIS infrastructure undermined evidence-based decision-making in PHC^54,55^. Governmental centers, being better integrated into the national HIS, delivered more reliable performance in data collection and reporting. Strengthening HIS through interoperable, role-based platforms and streamlined reporting mechanisms is therefore critical for both models. Service delivery gaps further compounded these challenges. Outsourced centers often lacked on-site laboratories and pharmacies, which fragmented patient care pathways, while incomplete referral and counter-referral systems weakened continuity of care. Similar shortcomings have been documented in Sri Lanka and Brazil, where partial integration of PPPs into PHC frameworks reduced their effectiveness^56,57^. Participants also highlighted cultural barriers, low health literacy, and financial constraints that limited access to NCD services. Although governmental centers were not immune, they were better positioned to adapt services to local community needs. International evidence emphasizes that PPPs can only be effective if they go beyond service expansion to incorporate cultural adaptation, equity, and patient-centered design.

Financing and incentives emerged as another critical determinant. Outsourced centers faced delays in payments, the absence of performance-based financing, and a persistent misalignment between workload and compensation. These problems echo experiences from India and Pakistan, where irregular financing undermined staff motivation and service quality^50,53,54^. In contrast, Brazil’s Previne Brasil program demonstrates that performance-based financing can address gaps in PHC and NCD management^9^. Timely disbursements and pay-for-quality schemes are thus essential for PPP sustainability. Infrastructure and facility readiness represented additional obstacles: outsourced centers frequently lacked adequate diagnostic tools, sufficient physical space, and basic supplies such as glucose strips and blood pressure monitors, while governmental centers, though relatively better resourced, also experienced shortages. These challenges mirror conditions in Sub-Saharan Africa and South Asia, where facility readiness is widely recognized as a prerequisite for effective NCD care^56,58,59^. Sustained investment in infrastructure and supply chains is therefore indispensable for ensuring comprehensive and equitable care.

Collectively, the qualitative findings highlight governance gaps, workforce instability, HIS weaknesses, financing irregularities, and infrastructural shortcomings as systemic barriers that require urgent reform.

#### Comparative analysis of PPP models in LMICs

To contextualize the Iranian experience, we examine successful PPP models in other LMICs. In India, governance reforms under the National Health Mission emphasize clear contracts, accountability mechanisms, and institutional capacity to sustain PPP-driven PHC^60^. The Manipal model demonstrates how integrating service provision with workforce training can stabilize quality and expand access^61^. In Bangladesh, PPPs have expanded maternal and child health services, with success tied to robust oversight and performance-based financing. Pakistan’s Lady Health Worker program, though not strictly a PPP, illustrates how community-based models with stable financing achieve sustainability. In Brazil, the Family Health Strategy and Previne Brasil reforms show how PHC-oriented financing and performance-based incentives improve coverage and quality, though challenges in hypertension and diabetes management persist^9^. Evaluations from Bahia State highlight the role of political and financial dynamics in shaping PPP hospital contracts^62^. During COVID-19, Brazil’s PPPs adapted to provide medicines but remained vulnerable to systemic shocks^38^. These cases underscore that PPPs can enhance NCD management in LMICs when supported by strong governance, transparent financing, and integrated service delivery.

#### Integrated synthesis and policy implications

Our integrated findings reveal that governmental PHC centers deliver more consistent and sustainable NCD management, while outsourced centers face systemic vulnerabilities in governance, workforce, financing, HIS, service delivery, and infrastructure. The Iranian experience highlights both the potential and pitfalls of PPPs in LMIC health systems. While PPPs can expand access and drive innovation, they risk perpetuating fragility and inequity without structural reforms.

We propose several policy implications. First, governance reforms should prioritize contractual clarity, independent monitoring, and robust state stewardship. Second, workforce policies must address instability through comprehensive incentive packages, including job security and career development. Third, HIS requires interoperable platforms and streamlined reporting to support evidence-based decision-making. Fourth, financing mechanisms should ensure timely payments and reward quality and continuity over volume. Finally, sustained investment in facility readiness and supply chains is critical for effective NCD management.

These recommendations align with Sustainable Development Goal (SDG) 3.4, which aims to reduce premature NCD mortality, and the WHO Global NCD Action Plan, emphasizing PHC strengthening, intersectoral collaboration, and equitable financing. The Iranian case offers transferable lessons for LMICs scaling PPPs in NCD care: success hinges not on whether services are public or private, but on the design and implementation of systemic governance, financing, and accountability frameworks.

#### Limitations of the study

This study faced several limitations. First, we included only eight PHC centers (four governmental and four outsourced), which constrained statistical power and limited the generalizability of our findings. Second, socioeconomic and contextual differences among catchment populations may have influenced performance indicators, despite our efforts to standardize data. Third, recall bias may have affected the qualitative phase, as we conducted interviews in 2024 about activities from 2018 to 2022. Finally, the COVID-19 pandemic disrupted NCD services globally, and its impact on 2020–2021 data should be considered when interpreting trends.

## Conclusion

Our study shows that while public-private partnerships (PPPs) hold potential to enhance primary health care (PHC) capacity for NCD management in Iran, their effectiveness is hindered by systemic challenges in governance, workforce stability, Health Information Systems (HIS), and infrastructure. To address these issues, we recommend policy interventions such as standardized workforce contracts, timely remuneration mechanisms, performance-based dashboards for monitoring NCD indicators, and strengthened referral pathways. These targeted reforms, aligned with the WHO health system building blocks framework, can improve the sustainability and equity of PPP-driven NCD care in Iran and offer transferable lessons for other low- and middle-income countries (LMICs).

## Supplementary Information

Below is the link to the electronic supplementary material.

Supplementary Material 1

Supplementary Material 2

Supplementary Material 3

## Data Availability

All data generated or analysed during this study are included in this published article.

## Abbreviations

Abbreviation: Full term
NCD: Non-Communicable Disease
LMIC: Low- and Middle-Income Country
SDG: Sustainable Development Goal
PPP: Public–Private Partnership
PHC: Primary Health Care
CHC: Comprehensive Health Center
CHV: Community Health Visitor (community-level health staff)
HTP: Health Transformation Plan
IHS: Integrated Health System
KPI: Key Performance Indicator
WHO: World Health Organization
