# Supplementary material for: Assessing the performance of public–private partnerships in non-communicable disease management with a mixed-methods approach
Source: Sci Rep. 2025 Nov 11;15:39513. doi: 10.1038/s41598-025-23266-7 (PMC12606227; doi:10.1038/s41598-025-23266-7)
Supplement: Supplementary file 1 — Supplementary Material 1 [file 41598_2025_23266_MOESM1_ESM.docx]

**Interview Guide Questions**

Dear Expert, as a healthcare provider, please provide guidance on the following questions:

1- Please briefly introduce yourself.

2- What is your level of education?

3- How many years of experience do you have in the field of non-communicable disease care?

4- Please describe the process of implementing non-communicable disease programs (diabetes, hypertension, blood lipids, cardiovascular disease risk assessment)?

5- Please tell me how you assess the status of non-communicable disease indicators (diabetes, high blood pressure, blood lipids, cardiovascular disease risk assessment)? (If not meeting expectations, explain the reasons: various reasons including structural problems, human resources, payment system, training, supervision, etc.)

6- Please tell me what challenges you think exist in the proper implementation of the program for prevention, screening, diagnosis, care, and follow-up of non-communicable diseases (diabetes, high blood pressure, blood lipids, cardiovascular disease risk assessment) at the primary health care level in Hormozgan province?

7- Please tell me what solutions you suggest to improve the management process of implementing non-communicable programs (diabetes, high blood pressure, blood lipids, cardiovascular disease risk assessment)?

* Probing questions for further investigation (for example, can you explain more? Can you give an example?) will also be asked during each interview session.
