# Supplementary material for: Assessing the performance of public–private partnerships in non-communicable disease management with a mixed-methods approach
Source: Sci Rep. 2025 Nov 11;15:39513. doi: 10.1038/s41598-025-23266-7 (PMC12606227; doi:10.1038/s41598-025-23266-7)
Supplement: Supplementary file 3 — Supplementary Material 3 [file 41598_2025_23266_MOESM3_ESM.docx]

**Supplementary Tables S1–S4.** Independent-samples t-test results for diabetes, hypertension, dyslipidemia, and cardiovascular risk indicators

Additional inferential analyses were conducted using independent-samples t-tests comparing governmental and outsourced CHCs. Results are summarized below. No statistically significant differences were observed (all p>0.05).

Supplementary Table S1. Diabetes detection (%): Independent-samples t-test results

| Year | Gov_mean | Out_mean | p_value |
| --- | --- | --- | --- |
| 2018 | 0.46 | 0.39 | 0.76 |
| 2019 | 0.19 | 0.26 | 0.45 |
| 2020 | 0.15 | 0.24 | 0.44 |
| 2021 | 0.07 | 0.11 | 0.58 |
| 2022 | 0.11 | 0.07 | 0.55 |

Supplementary Table S2. Diabetes control (%): Independent-samples t-test results

| Year | Gov_mean | Out_mean | p_value |
| --- | --- | --- | --- |
| 2018 | 51.69 | 45.09 | 0.81 |
| 2019 | 57.27 | 44.82 | 0.28 |
| 2020 | 62.59 | 87.18 | 0.21 |
| 2021 | 47.29 | 75.00 | 0.45 |
| 2022 | 55.39 | 40.32 | 0.57 |

Supplementary Table S3. Hypertension detection (%): Independent-samples t-test results

| Year | Gov_mean | Out_mean | p_value |
| --- | --- | --- | --- |
| 2018 | 0.71 | 0.64 | 0.81 |
| 2019 | 0.45 | 0.57 | 0.50 |
| 2020 | 0.23 | 0.27 | 0.79 |
| 2021 | 0.11 | 0.13 | 0.81 |
| 2022 | 0.15 | 0.12 | 0.85 |

Supplementary Table S4. Dyslipidemia prevalence (%): Independent-samples t-test results

| Year | Gov_mean | Out_mean | p_value |
| --- | --- | --- | --- |
| 2018 | 8.11 | 7.56 | 0.86 |
| 2019 | 8.09 | 6.89 | 0.70 |
| 2020 | 8.16 | 4.91 | 0.21 |
| 2021 | 16.67 | 4.44 | 0.19 |
| 2022 | 15.03 | 4.50 | 0.10 |
